# Two-dimensional Wrinkle Resonators for Random Lasing in Organic Glasses

**DOI:** 10.1038/s41598-020-59236-4

**Published:** 2020-02-12

**Authors:** Nicolai M. Hoinka, Christoph Ostwald, Thomas Fuhrmann-Lieker

**Affiliations:** Macromolecular Chemistry and Molecular Materials, Institute of Chemistry and Center for Interdisciplinary Nanostructure Science and Technology, University of Kassel, Heinrich-Plett Str. 40, 34132 Kassel, Germany

**Keywords:** Materials chemistry, Physical chemistry, Materials for optics, Lasers, LEDs and light sources, Optical physics

## Abstract

Random lasers consisting of slab waveguides with two-dimensional disordered wrinkling patterns that act as scattering resonators are reported. As active material 2,2′,7,7′-tetraphenyl-9,9′-spirobifluorene is used which is sandwiched between an oxidized silicon wafer and a cladding with higher glass transition temperature. Wrinkles with tailorable periodicity have been induced by thermal annealing. Photopumping experiments show the transition from amplified spontaneous emission to a multiple peak laser spectrum with linewidths as low as 0.1 nm, demonstrating the applicability of this approach for random laser design.

## Introduction

The phenomenon of random lasing has resurrected great interest recently, after its theoretical postulation^1^ and experimental realization in a variety of systems^2–6^, including dye containing liquids^7,8^, powders^2,9^, scattering enhancing liquid crystals^10,11^ and especially polymer-based dye-doped waveguide materials^6,12–16^. Polymers provide flexible, low cost materials with a spectral range only limited by the gain material used as dopant.

Random lasing is based on amplified emission and multiple scattering in disordered media and, in literature, it is classified depending on the present feedback mechanism in either incoherent or coherent random lasing. The latter results in sharp lines based on resonant feedback loops and exist both in strongly scattering, localized regimes and in weakly scattering, diffusive regimes. Incoherent random lasers, or non-resonant feedback lasers are terms that represent a variety of light amplifications in disordered media, but they have neglectable feedback and result in a smooth, broader amplification peaks (several nm). In organic photonics, thin waveguide structures are popular for lasing devices due to easy manufacturing techniques and their compatibility with integrated optics^17–19^. The confinement that is introduced by the waveguide structure is often sufficient for non-resonant stimulated emission in organic laser dyes called ASE or “travelling wave lasing” without the necessity of additional confinement by a resonator, when gain is introduced. A key aspect is that the gain length needs to be large enough to exceed the losses which defines the threshold of ASE in waveguiding structures^20,21^. Similar to non-resonant feedback lasing, it results in drastic spectral narrowing for fluences above threshold. Consider a waveguide structure in an intermediate regime between slight disturbances on the surface due to increased roughness and increasing backscattering in analogy to quasi-waveguides which show losses introduced by uneven substrates. Here, the modes can still travel through the sample only that scattering at the surface is introduced. Such lossy, asymmetric slab guide systems have already been reported^13,15,16^ and light amplification might be introduced due to both multiple scattering and waveguiding in a still confined, but lossy slab guide structure. For the non-resonant case, we refer to this type of emission as amplified spontaneous emission and we make a clear distinction to resonant random lasing.

**Figure 1:**
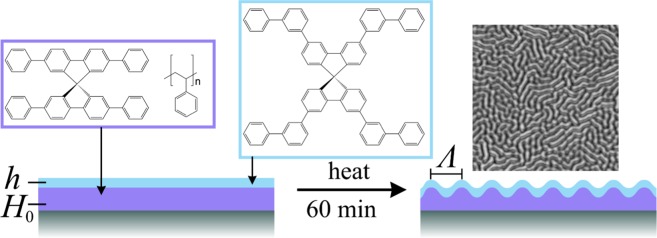
General scheme for thermally induced wrinkling in a layer system consisting of an oxidized silicon wafer as substrate, Spiroquaterphenyl in polystyrene as central layer, and *m,m*-Spirosexiphenyl as cladding.

Random scattering has been introduced into organic waveguides by dopants^22–25^, by surface roughness^16,25–27^, and by one-dimensional wrinkles in polydimethylsiloxane (PDMS) substrates^28,29^. Here, we apply self-organized wrinkling patterns with a characteristic two-dimensional morphology. Such wrinkles can be induced in multilayer systems consisting of a liquid or viscoelastic core covered by an elastic cladding. They can be found on the human skin as well as on Saturn’s moon *Enceladus* in which they indicate the presence of liquid water under an ice layer^30^. On a smaller device scale, they can be realized for example with deformable PDMS substrates^31,32^ or with organic glasses between a substrate and a cladding layer^33–35^. They have in common that intrinsic stresses relax by amplification of fluctuations of a certain wavelength range, giving rise to the typical two-dimensional morphology. Resembling spinodal patterns in soft matter self-organization, the surface corrugation process is sometimes referred to as “spinodal wrinkling”^33,34^. The spatial wavelength *Λ* of a wrinkled surfaced is defined as the average peak to peak distance (see Fig. 1). The relaxation is induced mostly by thermal treatment, i.e. for organic glasses or polymers by heating the central layer above glass transition temperature whereas the cladding layer remains solid and prevents spinodal dewetting and thus, makes thermal wrinkling possible. Also, photoinduced approaches have been reported^36–38^. The morphology, the spatial wavelength *Λ* and the dynamics are controlled by the mechanical properties of the materials, the film thicknesses, intrinsic stresses due to the preparation as well as by the treatment for relaxation. Tailoring disordered wrinkling has proven to be of great potential for optoelectronic materials^39–41^ and sensorics^40^.

Thus, in this contribution we combine the technique of two-dimensional wrinkling with the use of highly efficient organic solid-state laser materials, namely spiro oligophenyls that have been reported as low-threshold laser materials with high thermal stability^42–45^. As central and emissive layer, polystyrene doped with the active dye 2,2′,7,7′-Tetraphenyl-9,9′-spirobifluorene (Spiroquarterphenyl) was selected. As cladding, 3,3′,6,6′-Tetrakis-(biphenyl-3-yl)-9,9′-spirobifluorene (*m*,*m*-Spirosexiphenyl) was used that has a higher glass transition temperature (*T*_g_ = 140 °C) as polystyrene and does not show any significant absorption at the wavelength of the pumping laser^45^. Bilayer samples with a total thickness below 250 nm were prepared on oxidized silicon wafers as substrate. By thermal treatment, wrinkles were induced due to the stress at the interfaces (Fig. 1) and characterized. Then, by pumping with light pulses at 337 nm, the emission behavior of the samples was studied for random lasing.

## Results and Discussion

In our experiments, as matrix in the central layer polystyrene charges of two different molecular weights are used that differ significantly in glass transition temperature (70 °C and 110 °C), but not in their optical properties, as confirmed by variable angle spectroscopic ellipsometry. For both matrices, the thickness *H*_0_ of the active central layer as well as the thickness *h* of the *m,m*-Spirosexiphenyl cladding was varied. In all cases, annealing above the glass transition temperature of the matrix resulted in the formation of wrinkles with average periodicities *Λ* (spatial wavelength), determined from atomic force microscopy (AFM) images via Fourier transform techniques (see experimental section). The reason two different matrices were chosen was to investigate the influence of the molecular weight on such a system. However, it turns out that the thickness dependencies and similar viscosities at the respective annealing temperatures are more dominant factors for the appearance of wrinkles. The full set of samples is given in the supplementary information, showing that as expected the winkles have larger periodicities at larger thicknesses, with statistical deviations and no clear dependence on the molecular weight of the polystyrene due to similar viscosity at the respective annealing temperatures.

**Figure 2:**
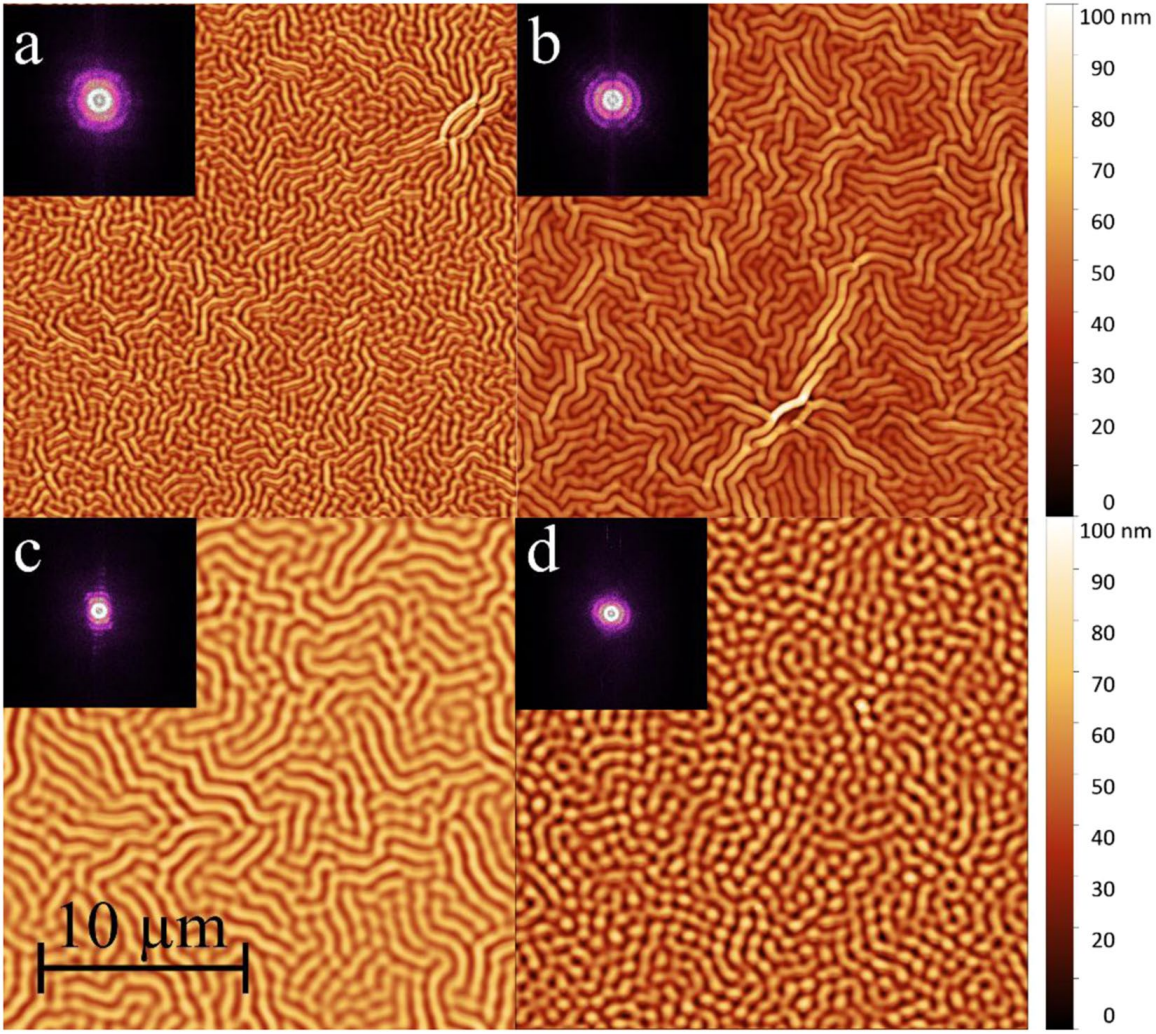
Wrinkle structures measured by atomic force microscopy for the analyzed samples after 60 min of annealing. The letters *a* to *d* correspond to the sample’s names. The scale bar as well as the height scale applies to all four images. As an inset, the respective Fourier transformations are shown.

In a more detailed analysis, we shall concentrate on four samples from the series of the polystyrene with lower *T*_g_ and pairwise similar values for *H*_0_ and *h*. Within uncertainties in the individual preparation, sample *a* and *c* as well as *b* and *d* have similar thicknesses of the central layer (~101 nm and ~173 nm), whereas *a* and *b* as well as *c* and *d* have common thicknesses of the cladding layer (~11 nm and ~23 nm), respectively. The numbering corresponds to the samples listed in Table 1 in which detailed analytical data are compiled. In Fig. 2, the AFM surface images of these samples after an annealing time of 60 minutes are displayed. For better comparison, the height scale is kept equal in every image and the scale bar in Fig. 2 applies for all four samples. The Fourier transformations of the images are shown as insets. Samples *a* to *c* show the typical large, parallel shaped labyrinthine pattern that seem to be periodic in small areas and indicate an advanced state of wrinkling where the most stable wavelength^46^ is dominant. Sample *d* shows a rather island like structure, although the formation of a labyrinth pattern is already visible. This describes the early state of wrinkle formation that all four samples went through^34^. This sample exhibits the largest film thickness, leading to a delayed wrinkle formation. A longer exposure would also reveal the labyrinthine structure more clearly, but for the sake of comparison the annealing times have been harmonized. During the transition from the fastest growing to the most stable spatial modes, coarsening occurs^33^, therefore it is not surprising that *Λ* of sample *d* is still in the same range as for samples *b* and *c*, whereas sample *a* exhibits a significantly smaller pattern (values are given in Table 1). The height modulation Δ*H* in the wrinkles is in the order of 10–20% of the total thickness.

**Table 1:** Overview of the samples discussed in the text. *H*_0_: thickness of the central layer, *h*: thickness of the cover layer, *n*_H0_, *n*_h_ refractive indices of the respective layers at 400 nm, *Λ* spatial wavelength as mean peak-to-peak distance, Δ*H* modulation depth, *n*_eff_, effective mode index, *γ* gain factor as described in the text.

Sample	*H*_0_/nm	*h*/nm	*n(H)*	*n(h)*	*Λ*/nm	Δ*H*/nm	*n*_eff_	*γ*/*%*
***a***	101.2	11.0	1.635	1.826	567	11.3 ± 3.0	1.486	34.2
***b***	176.4	11.1	1.646	1.823	840	12.1 ± 4.6	1.534	53.2
***c***	101.7	21.5	1.632	1.851	851	24.6 ± 7.9	1.507	33.9
***d***	171.2	25.3	1.641	1.874	827	16.2 ± 4.6	1.544	45.8

Considering the optical properties of the layer system at the reference wavelength of 400 nm as the emission maximum of Spiroquaterphenyl, we can state that there is a waveguide formed by the two organic layers between the substrate with refractive index *n*(400 nm) = 1.475 and air (*n = *1.0). Due to the amorphous character of the films, the refractive indices may vary slightly, so they are determined individually for each sample. The cladding layer has with *n*~1.85 a higher refractive index relative to the central layer with *n*~1.64. This way the thickness of the cladding layer not only affects the effective refractive index of a mode in the waveguide, but more importantly these modes are drawn towards the surface, when *h* is increased. The upper limit for this effect is given by the cut-off thickness of the cladding layer (~46 nm).

**Figure 3:**
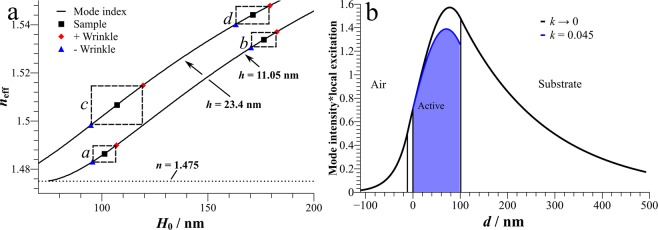
(**a)** Effective refractive index in dependence of the thickness of the central layer for two different thicknesses of the cladding. Indicated are the samples with their mean thickness (black squares) and thicknesses in the wrinkle valleys (blue triangles) and hills (red diamonds). For calculation, averaged cladding thicknesses are taken as 11.05 nm for *a* and *b*, and 23.4 nm for *c* and *d*. (**b**) Mode intensity profile for the TE_0_ mode of sample *a*. The relative contribution of the pump intensity profile to the emission into this mode according to the integral in Eq. (1) is indicated by the shaded area.

The waveguides are monomodal in each polarization for all samples, since the onset for a second waveguide mode occurs around thicknesses of 400 nm. The effective mode index is modulated by the wrinkles which is indicated in Fig. 3a by rectangles around the mode index of the flat films. As the mass transport only takes place in the dye-doped polymer layer, the modulation in the effective refractive index is attributed to this effect. The cladding layer stays approximately the same. This effective index modulation causes scattering in the waveguide modes and is the key factor for possible feedback in theses samples. For example, *c* exhibits the largest thickness profile leading to a modulation in effective index by Δ*n*_eff_ = 0.017. Since the modulation is not too high, the system can be regarded in first approximation as a two-dimensional disordered landscape of effective indices in analogy to distributed feedback systems but with randomly distributed disorder^47^. If in the valleys of the wrinkles the mode index would fall below the cut-off thickness, the wrinkles would consist of optically isolated waveguides only coupled by evanescent fields, but this is obviously not the case.

The effectivity of optical pumping depends on the penetration depth. We follow the approach given in ref. ^48^ and calculate the overlap of the waveguide mode with the depth-dependent excitation density in form of a gain factor

1 $$\gamma =\frac{{n}_{active}}{{n}_{eff}}\frac{{\int }_{active}{E}_{y}^{2}\cdot \text{exp}(-{\alpha }_{ex}x)\text{d}x}{\int {E}_{y}^{2}\text{d}x}$$

in which *n*_active_ is the refractive index of the active layer, *n*_eff_ the mode index, *E*_y_(x) the electric field profile of the TE mode and α_ex_ the attenuation coefficient at the pumping wavelength of 337 nm. With an extinction coefficient *k = *0.045 as imaginary part of the refractive index, α becomes 1.7·10^-3^ nm^−1^ and is sufficiently low that the entire active zone is pumped almost homogeneously. There is a trade-off between mode profile and penetration depth, but it turns out that here thicker films improve the gain factor because per area more light can be absorbed. The overlap integral is shown in Fig. 3b as blue area for illustration.

**Figure 4:**
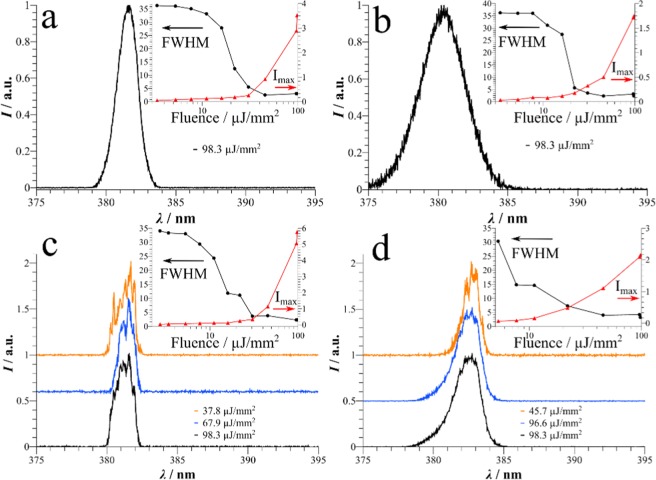
Normalized amplified emission of the analyzed samples. While samples (**a** and **b**) only achieve ASE, samples (**c** and **d**) show resonant random lasing at various fluences. For better illustration, the baselines for different pumping fluences are shifted in (**c** and **d**). Each inset shows a plot of the respective FWHM (in nm) and maximum intensity *I*_max_ versus the fluence. The lines combining two data points are used for better illustration.

In Fig. 4, emission spectra of all four samples *a* to *d* under pulsed excitation conditions at a fluence up to the order of 100 µJ/mm^2^ are plotted. All samples were analyzed at least at 3 randomly chosen spots. Samples *a* and *b* with the thinner cladding show emission lines characteristic for ASE with full width at half maximum (FWHM) of 1.74 nm and 3.78 nm, respectively. Lowering or increasing the incident fluence or the analyzed area of illumination did not change the shape of the spectrum, provided that the threshold for amplified spontaneous emission is exceeded. Since a spectrum of non-wrinkled Spiroquarterphenyl doped polystyrene under the same measurement conditions exhibits a better signal-to-noise ratio (see Figure S1), these spectra indicate that the wave-guiding ability of the samples is clearly disturbed, and wrinkling does have an impact on the optical analysis of the samples. Still, both FWHM are significantly lower that the full width at half maximum of the fluorescence at 36 nm. A plot displaying the narrowing with increasing fluence in comparison to fluorescence for a smooth dye-doped polystyrene layer is shown in Figure S2.

In the spectra of samples *c* and *d*, on the other hand, sharp narrow lines appear which can clearly be distinguished from noise since their relative intensity varies with the excitation fluence. At lower fluence, more lines can be resolved. We assign these to distinct confined, but lossy resonant random laser modes in the disordered structure with rather low Q-factors. At higher fluences more modes are excited, which leads to a broadening of the overall spectrum. Here, the determination of distinct modes becomes difficult. The insets in Fig. 4 show the decrease of the FWHM (in nm), as well as an increase of the peak intensity (in a.u.) with increasing fluence for each sample. We stress out that the line in between two data points in the insets are only used for better illustration. Regardless of origin of the amplification, all four samples have a threshold where a drastic reduction of the FWHM and a change in slope for the output intensity is detectable. The thresholds of the samples vary between estimated 30 µJ/mm^2^ for samples *a*, *b* and *c* to 10 µJ/mm^2^ for sample *d*. Especially for samples *c* and *d* the threshold depends on the quality of the cavity for these resonant random lasing modes. Thus, one can say that the losses for resonant modes excited in sample *d* are reduced compared to *c*.

**Figure 5:**
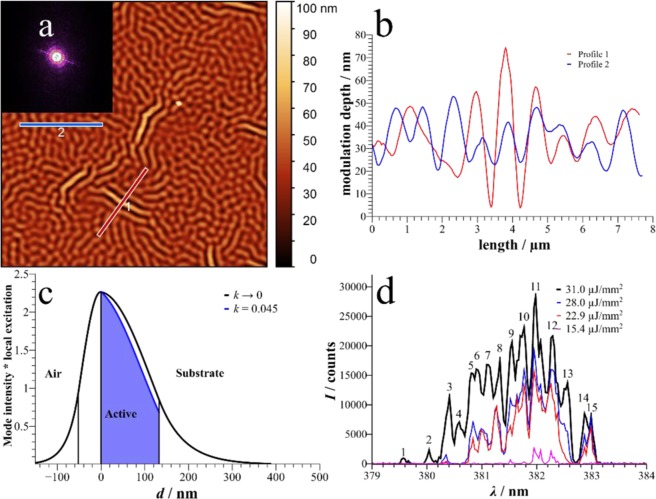
(**a)** Morphology of a sample from a series with polystyrene of higher T_g_. (**b)** Profile of the cross section indicated in a). (**c)** Mode and excitation profile of this sample as in Fig. 3b). (**d)** Line spectrum at different pump fluences indicating well defined modes.

One may ask whether the optical path between two wrinkles is commensurate with the emission wavelength *λ*_em_ of the resonant random laser modes in order to produce Bragg-like resonances. However, such a correlation does not exist, so the resonator mechanism is more complicated and involves a larger part of the disordered structure. The product of effective index and wrinkle periodicity gives 2.2 *λ*_em_ for sample *a* and approximately 3.3 *λ*_em_ for the other three samples in Fig. 2. Considering all measured samples, the occurring of single modes in the spectrum and therefore resonant random lasing cannot be predicted from the layer thicknesses and resulting periodicity alone. It rather depends on the morphology of each specimen and part of the sample that is irradiated. As a rule of thumb, Λ ≈ 850 nm shows a higher probability for the detection of resonant random laser modes. Or more general: the prediction of the feedback structure is not possible, but merely the average periodicity. It can be surmised, however, that a high modulation depth favors resonant modes due to the larger amount of scattering. In the series with polystyrene of the higher *T*_g_ only two samples showed this behavior, with a similar thickness range, up to even higher thicknesses of the cladding. The results for one of the samples are shown in Fig. 5. The layer thicknesses are *H*_0_ = 134.0 nm and *h* = 50.2 nm, resulting in *n*_eff_ = 1.576 and *Λ* = 1144 nm. Again, optical path and emitting wavelength are not commensurate (*n*_eff_ *Λ* ≈ 4.5 *λ*_em_). The average modulation depth is 21.0 nm. Figure 5a shows the AFM measurement in which areas with established wrinkle formation and higher modulation depth are visible, while in most of the sample island-like structures are expressed. The respective profiles are shown in Fig. 5b. Due to the increased thickness of the cladding layer with respect to samples *a* to *d*, the major part of the mode is guided within the organic layers (Fig. 5c) which results in *γ* = 55.0% (blue filled area). Here, in the emission spectrum no ASE can be seen, but instead narrow modes with at least 15 established peaks arise (Fig. 5d). This represents the clearest evidence for resonant random laser modes in our samples, and the linewidth can easily be deduced as being in the range of 0.1 nm. The evolution of distinct modes with higher losses can be seen e.g. for modes 2 and 4, as they arise only at larger fluences.

In all shown spectra, the aspect of bleaching needs to be considered. A reduction of the peak intensity by half was already achieved after roughly 150 pulses with 97.8 µJ/mm^2^. Figure S3 shows the respective plot against the number of pulses. However, the FWHM increased with a delay between 300 pulses and 400 pulses. By encapsulating the devices to either measure under vacuum conditions or in an inert atmosphere, the reduction in intensity by degradation effects can be slowed down significantly^48^. Also, these wrinkled thin films are simple to produce under moderate conditions and it is easy to replace a bleached region by shifting the sample spatially to a pristine region, of course at the cost of selecting different spectral modes. Independent of an envisaged application of such a random laser, we think our new findings will contribute to a better understanding of the mode development in disordered structures.

In conclusion, we demonstrated that bilayers of dye-doped polystyrene, capped with a layer of higher *T*_g_ material are capable of resonant random lasing with narrow linewidths if the surface is wrinkled by thermal treatment. Suitable wrinkle periodicities for this process are around 850 nm or 1150 nm. Deviation from the optimum structure causes broader lines of amplified spontaneous emission which presumably represent non-resonant, travelling waves. Due to the statistical process in wrinkle formation, predictions for the spectrum are still difficult, and it remains to be shown how these modes overlap in their lateral extension.

## Experimental Section

### Sample preparation

The spiro-based waveguide samples were fabricated by a combination of spin coating and physical vapor deposition (PVD) on top of an oxidized silicon wafer with a 300 nm layer of silicon oxide. For spincoating, 50 mg polystyrene with either an average molecular weight of 35,000 g/mol (*T*_g_ = 70 °C, Sigma Aldrich) or self-produced polystyrene (*T*_g_ = 110 °C) and 5 wt.% Spiroquarterphenyl were dissolved in 3 ml chloroform (Merck Uvasol). Depending on the desired thickness, the rotation speed of the self-built spincoater was varied between 890 rpm and 1900 rpm. As a cladding layer, 3,3′,6,6′-Tetrakis-(biphenyl-3-yl)-9,9′-spirobifluorene (*m*, *m*-Spirosexiphenyl) was evaporated by PVD in a vacuum chamber (Bestec) at pressures of about 1·10^-7^ mbar and lower. Layer thicknesses were determined after each step by ellipsometry (see below). Thermal wrinkling was induced by heating the samples to 5 K above the glass transition temperature (i.e. 75 °C and 115 °C, respectively) and annealing for 60 min. An overview of all prepared samples is given in Tables S1 and S2.

### Analytical methods

Glass transition temperatures of the materials as powder were determined by differential scanning calorimetry (Perkin Elmer DSC 7).

Layer thicknesses, refractive index dispersion as well as extinction coefficients were obtained with a spectroscopic VASE ellipsometer (J. A. Woollam) by measuring at least at three different angles (65°, 70°,75°) and in a spectral range from 300 nm to 1500 nm in steps of 5 nm. Silicon as well as the silicon dioxide layer was modeled according to ref. ^49^ For the determination of the organic layer thicknesses Cauchy models were applied, using the experimental data in the non-absorbing range higher than 400 nm.

AFM measurements were performed with a Nanowizard 2 (JPK BioAFM, Bruker) in intermediate contact mode. The line scan frequency was adapted individually. AFM images were evaluated with *Gwyddion* 2.4. For leveling, a third-degree polynomial function was applied. To determine the average peak to peak distance *Λ* of the samples, a self-written program in python was used. After Fourier transformation of the respective images, the sum over the radius in k-space is calculated and fitted assuming a gaussian distribution whereby the reciprocal peak value corresponds to *Λ*. For the modulation depth Δ*H*, 5 random profiles through the samples are created and an average of the modulation depth of at least 70 wrinkles is calculated.

Optical experiments were carried out by using an LTB MSG 800 nitrogen laser (*λ* = 337.1 nm) with a pulse duration lower than 500 ps and a pulse frequency of 10 Hz. The intensity was adjusted by a continuous neutral density filter wheel. The focus area of the excitation beam was varied between 2.1 mm^2^ (Fig. 4) and 6.8 mm^2^ (Fig. 5d). The emitted light was collected at a 45° angle and analyzed by a detector array spectrometer (Avantes 3648) with a spectral resolution of 0.04 nm and the integration time was set individually for each sample. It varied from 3 s to 25 s.

## Supplementary information

Supplementary Information.
