# Supplementary material for: Two-dimensional Wrinkle Resonators for Random Lasing in Organic Glasses
Source: Sci Rep. 2020 Feb 12;10:2434. doi: 10.1038/s41598-020-59236-4 (PMC7015940; doi:10.1038/s41598-020-59236-4)
Supplement: Supplementary file 1 — Supplementary Information. [file 41598_2020_59236_MOESM1_ESM.pdf]

Supplementary Information

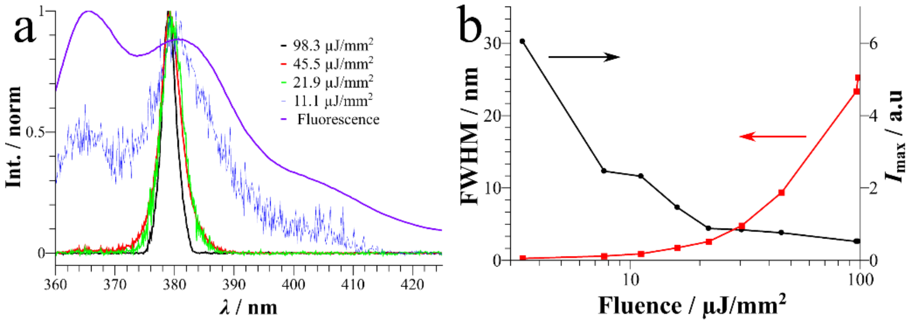

Figure S1. a) Spectral narrowing of 5% Spiroquarterphenyl in polystyrene (35.000 g/mol) in a thin wave guide film with an approx. thickness of 100 nm. A plot of the FWHM and the peak intensity vs. fluence is shown in b). The lines linking two data points are used for better illustration.

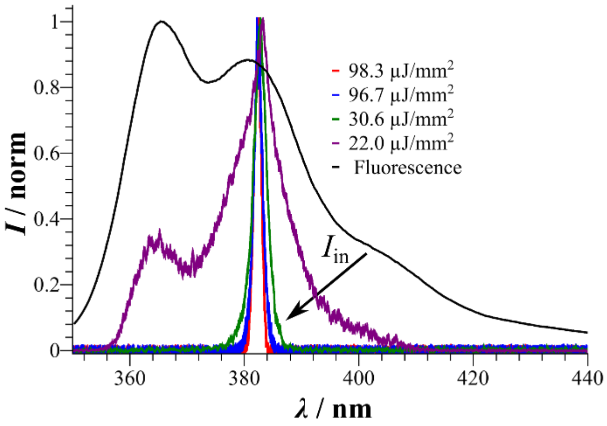

Figure S2. Narrowing FWHM with increasing input intensity for sample *d*. The black line denotes the fluorescence spectrum with $\lambda_{abs} = 337$ nm.

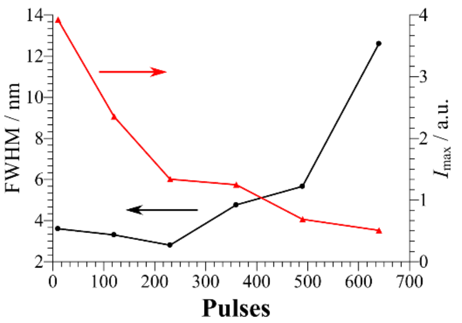

Figure S3. Plot of the full width at half maximum and the peak intensity vs the number of pulses. Roughly after 150 pulses with 97.8 µJ/mm^2 the peak intensity reduces down to half its initial value. However, an increase in FWHM is delayed and increases between 300 pulses and 400 pulses. The lines linking two data points are used for better illustration.

Table S1. Summarized parameters of analysed samples using polystyrene with a T_g =70 °C. The last row indicates whether ASE or resonant random lasing (RL) was detected in the optical spectra.

**Sample Nr.**	H_0 **/ nm**	***h*** **/ nm**	Λ **/ nm**	**RL/ASE**
a	101.2	11.0	567	ASE
b	176.4	11.1	840	ASE
**c**	**101.7**	**21.5**	**851**	**RL**
**d**	**171.2**	**25.3**	**827**	**RL**
1	230.9	28.3	99.7	ASE
**2**	180.6	21.9	1373	ASE
3	177.7	13.0	769	ASE
4	140.2	26.8	937	ASE
5	118.8	22.4	740	ASE
6	106.6	22.8	887	ASE
**7**	**104.7**	**22.2**	**817**	**RL**
**8**	**104.3**	**21.2**	**845**	**RL**
**9**	**104.1**	**22.2**	**760**	**RL**
10	102.8	11.2	617	ASE
11	93.0	21.8	842	-

Table S2. Summarized parameters of analysed samples using polystyrene with a T_g =110 °C. The last row indicates whether ASE or resonant random lasing (RL) was detected in the optical spectra.

Sample Nr.	H_0 / nm	h / nm	Λ / nm	RL/ASE
1	140.6	57.0	1313	ASE
2	140.5	65.6	1446	ASE
**3**	**138.0**	**44.7**	**1141**	**RL**
4	136.6	38.7	995	ASE
5	136.0	27.0	612	ASE
**6**	**134.4**	**50.3**	**1144**	**RL**
7	133.2	21.0	604	ASE
8	128.5	74.1	1542	ASE
